# Identification of Novel IGF1R Kinase Inhibitors by Molecular Modeling and High-Throughput Screening

**Published:** 2013

**Authors:** R. Moriev, O. Vasylchenko, M. Platonov, O. Grygorenko, K. Volkova, S. Zozulya

**Affiliations:** Enamine Ltd, Chervonotkatska Str., 78, Kyiv, Ukraine, 02094; Kyiv National Taras Shevchenko University, Volodymyrska Str., 64, Kyiv, Ukraine, 01601

**Keywords:** IGF1 receptor, tyrosine kinase inhibitor, anti-cancer drug candidate, high-throughput screening, virtual screening

## Abstract

The aim of this study was to identify small molecule compounds that inhibit the kinase activity of the IGF1 receptor and represent novel chemical scaffolds, which can be potentially exploited to develop drug candidates that are superior to the existing experimental anti-IGF1R therapeuticals. To this end, targeted compound libraries were produced by virtual screening using molecular modeling and docking strategies, as well as the ligand-based pharmacophore model. High-throughput screening of the resulting compound sets in a biochemical kinase inhibition assay allowed us to identify several novel chemotypes that represent attractive starting points for the development of advanced IGF1R inhibitory compounds.

## INTRODUCTION

The receptor of insulin-like growth factor type 1 (IGF1R) is a transmembrane receptor tyrosine kinase (RT K) widely expressed in various cell types and the tissues of all vertebrates. IGF1R is the key biological regulator of cell growth and survival, both in the developmental and adult states. The receptor is a very close phylogenetic relative of the insulin receptor (InsR), the major regulator of carbohydrate homeostasis, as well as lipid and protein metabolism. IGF1R shares almost 60% overall homology with InsR; the similarity is much higher (~ 90%) in the catalytic domain area of the receptors. The IGF pathway is commonly dysregulated in many human cancers, including breast, prostate, liver, lung, bladder, thyroid, renal cancers, Ewing’s sarcomas, rhabdomyosarcoma, lymphomas, leukemias, multiple myeloma, etc., primarily via increased expression of IGF1R or its ligands, IGF- 1 and IGF-2, and autocrine loops [1, 2, 3]. The IGF-1 receptor is needed for the transformation of cells by oncogenes; enhanced IGF-1 receptor expression can cause ligand-dependent, malignant transformation and tumorigenesis [4]. Mutated, constitutively upregulated forms of IGF1R kinase as cancer drivers have not been documented in the literature, contrary to the paradigm for oncogenic tyrosine kinases. The general anti-proliferative and pro-apoptotic effects associated with IGF1R inhibition, as well as the broad expression of IGF1R in tumors, are suggestive of a high clinical potential for IGF1R inhibitors in combination therapies. Because of the broad malignant neoplasia linkage to the ubiquitous IGF signaling pathway, therapeutic strategies that inhibit the IGF1R receptor using either small-molecule kinase inhibitors (TKIs) or monoclonal antibodies (mABs) have been actively explored in various types of cancers by a large number of pharmaceutical and biotechnology companies over the past 10–15 years. At least a dozen IGF1R inhibitors, both small-molecules and antibodies, are currently in late preclinical or clinical development.

Due to the very high degree of homology among the catalytic domains of IGF1R and InsR RT Ks, all of the known advanced IGF1R-targeting TKIs inhibit InsR to a significant degree, as well. As a result, these TKIs obviously could impair glucose homeostasis and lead to hyperglycemia and the concomitant diabetic complications in TKI-treated patients. Indeed, such hyperglycemic effects have been observed in pre-clinical models and, more recently, in the clinical trials of smallmolecule IGF1R inhibitors, casting some doubt on the perspectives for their long-term clinical development and therapeutic use. Over the past decade, the obvious selectivity problem associated with small molecules has led to a shift in interest towards the development of an intrinsically, highly selective monoclonal antibody or protein-based IGF1R blockers which target either the receptor itself or its ligands. However, due to the peculiarities of IGF signaling axis biology and the resulting substantial cross-talk between IGF1R and InsR-driven signaling, some degree of InsR co-inhibition is believed to be beneficial in oncology settings by most experts [5, 6]. On the other hand, the concept of precisely blocking IGF1R signaling by pharmacological agents that are highly selective at the molecular level turns out to be a gross oversimplification when applied to the actual systemic action of these agents *in vivo*. Both a lack of efficacy and hyperglycemic effects were found in the late pre-clinical and clinical studies of several advanced anti-IGF1R protein/antibody-based therapeutic candidates despite their ultimate molecular selectivity for the target [4, 7]. Some of the apparent underlying mechanisms of this non-selectivity *in vivo *are as follows: significant cross-activation of IGF1R receptors by insulin and vice versa; activity of anti-IGF1R antibodies on InsR-IGF1R heterodimers, compensatory mechanisms of the living organism, such as upregulation of InsR or induction of IGF1 and insulin biosynthesis upon depletion of IGF-1/IGF1R pools in the body [7, 8]. In addition to the selectivity aspects mentioned above, there are multiple mechanisms of resistance to IGF1R-targeted therapy, which might necessitate coinhibition to achieve efficacy [9].

Thus, despite the solid academic validation of IGF1R as a highly attractive drug target in oncology, as well as the sustained effort to develop therapeutically useful IGF1R pathway blockers of diverse molecular nature and mechanisms of action, so far the results of late-stage clinical trials remain less than exciting [7, 10]. This controversial and complicated landscape, in addition to the certain inherent advantages of small molecule drugs over antibody/protein-based therapeutics, creates a persistent drive for a continued search for clinically superior chemical inhibitors of IGF1R. Such target compounds might differ from their predecessors by virtue of their different mechanisms of inhibition, more selective tissue distribution, co-inhibition of other targets, and altered pharmacodynamics or a better balanced selectivity for IGF1R versus InsR.

In this study, we report on the identification of several small molecule IGF1R inhibitors as a result of the screening of a focused library of 2,935 compounds generated by the combined use of pharmacophore- and target structure-based models. The compound series found represent novel chemotypes and are potentially developable into clinical IGF1R inhibitors with favorably altered properties as compared to existing ones.

## EXPERIMENTAL

**Reagents and materials**

All the reagents for screening, including ADP-Glo™ Kinase Assay (Cat. V9401), Kinase System kits for IGF1R (Cat. V3581), InsR (Cat. V9411), Met (Cat.V3361), Syk (Cat. V3801), and BTK (Cat. V2941) kinases, were obtained from Promega Corporation (Madison, WI, USA) and used according to the manufacturer’s recommendations. The reference kinase inhibitors PQ401 (Cat. P0113), AG538 (Cat. T7697), staurosporine (Cat. S5921), as well as poly(Glu4,Tyr1), sodium salt (Cat. P0275 ), and dimethyl sulfoxide (Cat. 41640), were all purchased from Sigma-Aldrich (St. Louis, MO, USA). The low volume, U-bottom, white NBS 384-well microplates (Cat. 3673) used for all luminescent assays were from Corning (Lowell, MA,USA), and robotic liquid handler 384-channel tips (Cat. 5316) were from Thermo Scientific/ Matrix (Hudson, NH, USA). The polypropylene 384-well and V-bottom plates (Cat. 784201) were purchased from Greiner Bio-One (Monroe, NC , USA), and the 96-well plates, from Matrix (Cat. 4271), or similar ones, were used for compound storage and dilutions. The reagents and buffers for robotic multichannel pipetting were kept in disposable modular reservoirs (Cat. N372790) from Beckman Coulter (Indianapolis, IN, USA).

All the compounds iteratively tested in this study were selected from the ~1,900,000 compound collection of Enamine, Ltd. (www.enamine.net, Kyiv, Ukraine) and supplied by Enamine’s library formatting facility as frozen 10 mM solutions in dimethyl sulfoxide (DMSO) in heat-sealed 96- or 384-well polypropylene plates.

**Molecular modeling and chemoinformatics**

All computations were done using the QXP/Flo+ software package developed by McMartin *et al*. [11]. We used the computer cluster configuration HPC Linux cluster (164 CPU cores in 5 nodes). All the manipulations with chemical structures and databases were conducted in the Instant JChem software (ChemAxon, software version 5.10.1).

**Screening equipment and data analysis**

Multi-well liquid dispensing for setting up assays was performed using either the robotic liquid handler PlateMate Plus or the manual electronic multichannel micropipettes Matrix Impact (Thermo Scientific, Hudson, NH, USA). High-throughput Screening (HTS), kinase selectivity, and dose-response (IC_50_) assays were read in the luminescence mode using the PolarStar Omega reader (BMG Labtech, Ortenberg, Germany) or M5 reader (Molecular Devices Corp., Sunnyvale, CA). IGF1R ADP-Glo data in relative luminescence units (RLU) were collected from the plate readers, and the percentage of activity (% Activity) was determined for each point as follows: % Activity = 100*(RLU sample – RLU no kinase control avg)/(RLU kinase control avg – RLU no kinase control avg). The screening data were processed and visualized using Microsoft Excel templates designed to calculate the inhibition values, the Z’-factor, and GraphPad Prism 5 (GraphPad Software, Inc., La Jolla, CA) for IC_50_ analysis. The doseresponse curves of Percent Activity were fit in Prism, using a sigmoidal variable slope fit with the maximum % activity and the minimum % activity fixed at 100% and 0%, respectively. The Z′ factor, a statistical measure of variability and reproducibility for HTS assays, was determined using the following formula: Z′ = 1-[3 × (SD_sample_+SD_contol_)/|M_sample_-M_control_|] [12], where SD denotes the standard deviation and M denotes the mean for the samples and controls, respectively. Prior to starting HTS, assay conditions were optimized and validated with regard to the maximum ATP turnover (never exceeding 20%), an acceptable signal-to-background ratio (“assay window”) of at least 6, an acceptable Z’-factor of at least 0.6, as well as day-to-day and plate-to-plate reproducibility of the screening data. All primary screening was performed at 20 μM compound concentrations, and some of the weak hits were subsequently re-tested at higher concentrations (40 or 80 μM) for confirmation. Statistically significant HTS hits in the primary screening were defined as those that produced a kinase activity signal at least three standard deviations lower than the mean of the assay plate run (not including the plate controls). Lineweaver- Burk plots were created in Excel or Prism using the standard algorithms [13]. All assay development and validation and high-throughput screening procedures were carried out according to the general guidelines as published on the U.S. National Chemical Genomics Center web site (NC GC Assay Guidance Manual and High-throughput Assay Guidance Criteria, http://www.ncbi.nlm.nih.gov/books/NBK53196/).

**High-throughput screening (HTS)**

IGF1R high throughput screening (HTS) assays using the IGF1R Kinase System and ADP-Glo readout system (Promega Corp.) were performed in a final volume of 7 μL per test compound using a 384-well small volume microplate format. All liquid dispensing was done using the PlateMate Plus robotic liquid handler. 3 μL aliquots of the enzyme/substrate mixture containing 1 μg of the IGF1Rtide peptide substrate and 4 ng of recombinant IGF1R kinase in a 0.66× assay buffer were transferred to the plate wells. The assay buffer (1×) consisted of 40 mM Tris chloride, 20 mM magnesium chloride, 0,1 mg/ml bovine serum albumin, 2 mM manganese chloride, and 250 μM dithiothreitol (DTT ). To add the tested compounds to the reaction, starting stocks of 10 mM compounds in dimethyl sulfoxide (DMSO) were diluted with DMSO to 2 mM. Next, 3 μL aliquots of 2 mM compound stock solutions in DMSO were transferred to 83 μL volumes of a 1× reaction buffer and thoroughly mixed. Aqueous compound solution aliquots of 2 μL were then transferred into the assay plate to get a final concentration of each compound of 20 μM in 1% DMSO. The plates were pre-incubated at 27°C for 10 min with gentle shaking (300 rpm). The assay was started by adding 2 μL of the ATP stock solution to the reaction mixture to achieve a final ATP concentration of 50 μM. After 1.5 h of gentle shaking (300 rpm) at 27°C, the ADP-Glo reagent (7 μL) and, after an additional 40 min, the detection reagent (14 μL) were added. After the final incubation for 20 min at 25°C, luminescence was read using a PolarStar Omega multimode plate reader at a gain setting of 3500 and integration time of 0.2 s.

The compounds were tested in quadruplicates or duplicates during the screening. One quadruplicate sample per plate of 2 μM final staurosporine was used as a control inhibitor sample. For the columns 1 and 2 of each 384-well plate, 3 μL of the 0.66× assay buffer, instead of the enzyme/substrate mixture, and DMSO- spiked 1× assay buffer without test-compounds (final 1% DMSO in the reaction mixture) were used to produce a positive (no kinase reaction) control. For the columns 23 and 24 of each plate, a DMSO-containing buffer without test-compounds (final 1% DMSO) was used during the compound addition step to produce a negative (no kinase inhibition) control. Prior to the dose-response and selectivity studies, all primary screening hits were confirmed by re-testing the single concentration point inhibition of IGF1R by compound solutions freshly prepared from solid compound stocks under the same conditions as described above (“confirmation from powders”).

**Dose-response curves (IC_50_) and kinase selectivity measurements**

Kinase selectivity assays with InsR (dose-response measurements) and Met, Syk and BTK kinases (single point compound concentrations) using the ADP-Glo readout system were run under optimized experimental conditions similar to the IGF1R assay with regard to the kinetic parameters of kinase reactions.

Dose-response and IC_50_ measurements for the confirmed screening hits were conducted for IGF1R and InsR kinases. The conditions were similar to the HTS conditions described above, except that the compounds were plated in 8-point curves serially diluted 1:2 from 100 μM top concentrations and with a 1% final DMSO throughout, in quadruplicates for each compound dilution point. The time of incubation of kinase reaction mixtures was 2.5 or 4.5 hours at 27°C in different experiments both for the insulin kinase receptor and IGF1R, the dithiotreitol (DTT ) concentration was 500 μM, and the amount of recombinant insulin receptor kinase was 2 ng per well. DTT concentration was elevated to enhance the stability of the enzymes during 4.5 h incubation experiments. Compounds were typically serially 2-fold diluted in pure DMSO starting from 10 mM down to 19.5 μM to produce the final concentrations ranging from 100 μM to 195 nM in a 1% DMSO-aqueous reaction buffer.

The amounts of the Met, Syk, and BTK enzymes and the incubation time were optimized to ensure an ATP conversion not higher than 20% in all cases. The typical assay window (signal/background) was 3–5 for all kinases. The final volumes of the Met, Syk, and BTK kinase assay reaction mixtures were 5 μL. Two μL of the enzyme solution in a 1× reaction buffer (6 ng Met, 8 ng BTK, 4 ng Syk), 1 μL of the compound solution in a 2× reaction buffer, the ATP and substrate mixture in a 0.5× reaction mixture were added sequentially. The composition of the 1× reaction buffer was 40 mM Tris, 20 mM magnesium chloride, 0.1 mg/ml bovine serum albumin, 500 μM DTT at pH 7.5 with 1% final concentration of the DMSO, 50 μM ATP, and 0.2 mg/ml Poly(Glu4, Tyr1) substrates. The buffer was supplemented with 2 mM manganese (II) chloride in the case of BTK kinase. Compounds were tested at 40 μM concentrations. In each experiment, 6 wells with the enzyme, but no added compounds, were used as negative inhibition controls; 6 wells without tested compounds and the enzyme and 6 wells with the enzyme and 0.5–1 μM staurosporine were used as positive inhibition controls. Pre-incubation of the reaction mixture with compounds prior to ATP addition was performed for 20 min at 25°C in all experiments. The incubation of the reaction mixture lasted for 25 min at 37°C. All compounds were tested in 4 to 6 repeats. Conditions of ADP detection were as follows: 40 min incubation with an ADP-Glo reagent, followed by 30 min with a detection reagent at 25°C. Luminescence was read using a BMG Polarstar Omega reader at a gain setting of 4095 and measurement time of 0.5 s.

**Measurements of the Michaelis–Menten kinetics**

Serial dilutions of ATP and substrate polypeptide poly(Glu4,Tyr1) were tested in IGF1R kinase assays with the inhibitors L1 and T4 to produce the kinetic data for Lineweaver-Burk plots. In the ATP competition measurements, solutions of ATP and the compounds being tested were subjected to twofold serial dilution. Compound L1 was tested at concentrations of 100, 50, 25, 12, and 0 μM in combination with eight ATP concentrations ranging from 519 to 4 μM. Compound T4 was tested at the same concentrations in combination with eight ATP concentrations ranging from 1 mM to 8 μM. IGF1Rtide at a concentration of 143 μg/ml was used as a peptide substrate in both cases. All concentration points were quadruplicated. The amount of IGF1R kinase was 1 ng per well, the DTT concentration was 500 μM, and the the kinase reaction was incubated for 4 h at 27°C. The range of ATP concentrations used for the plot was narrowed to 6 points for L1 to get the best fit.

In the substrate competition measurements, the poly(Glu4,Tyr1) substrate was titrated by twofold dilutions to obtain 8 concentrations from 0.9 to 114.3 μM, assuming the average molecular mass of the substrate to be 12.5 kDa. Three concentrations of two hit compounds – L1 (50, 25, 0 μM) and T5 (50, 12.5, 0 μM) – combined with 8 peptide concentrations were assayed; the ATP concentration was 250 μM for the peptide-competitive assay. The DTT concentration was 250 μM; the amount of the IGF1R enzyme was 2 ng per well. In order to build the plot, the range of the used peptide concentrations was narrowed to 5 points to get a linear fit. Prior to ATP addition, the reaction mixture with compounds was incubated for 20 min at 27°C in all the experiments.

## RESULTS AND DISCUSSION

**Virtual screening – Target-based selection**

The general concept of this study was to implement the “smart screening” strategy relying on the iterative physical screening of small, focused compound libraries selected from the vast off-the-shelf collection of ~1.9 million compounds at Enamine (www.enamine.net). The selection was based on ligand- and target-based virtual screening supplemented with knowledge of the published data on existing IGF1R inhibitors and the crystal structure of its kinase domain. Compounds containing potential toxicophoric and reactive structural fragments were removed using the medicinal chemistry filtering criteria described elsewhere [14]. Such an approach assists in the elaboration of new pharmacologically active compounds without resorting to random, large-scale screening of chemical diversity. The rationale was to deviate from the known IGF1R inhibitor chemotypes and from the paradigm of catalytic site binding and direct ATP competition. Several diverse* in silico *modeling approaches were used to generate mini-sets consisting of several hundred compounds each, which were subjected to experimental screening in a biochemical kinase assay. A total of approximately 4,000 compounds were screened as a result of this effort, including the “hit expansion” screens of active compound (“hit”) analogs. Two of the approaches used, which were based on screening of 2,935 molecules, led to the discovery of chemotypes with attractive properties and structural novelty (described below).

**Fig. 1:**
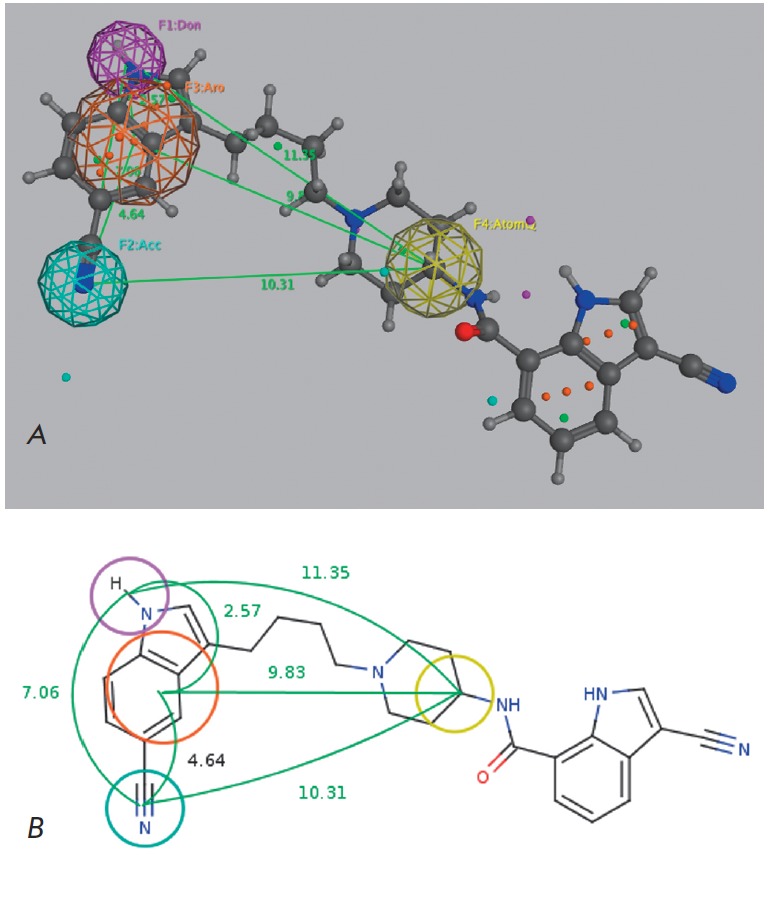
The pharmacophore model used for virtual library filtering. **A **— Pharmacophore model mapping of IGF1R inhibitor 3-cyano-N-{1-[4-(5-cyano-1H-indol-3-yl)butyl] piperidin-4-yl}-1H-indole-7-carboxamide derived from the ligand orientation in the crystal structure. The IGF1R inhibitor is shown in ball-stick representation. **B **— The generated pharmacophore model is shown with its interfeature distance constraints. Magenta — hydrogen bond donor; blue — hydrogen bond acceptor; orange — aromatic ring; yellow — any heavy atom; green — distances between the centers of the pharmacophore groups

A series of allosteric inhibitors of the IGF1R kinase domain have recently been reported [15]. The mechanism of action of these compounds is based on their binding to the allosteric protein surface pocket, which does not spatially overlap with the catalytic site and is located in the vicinity of the kinase domain activation loop that is triple-phosphorylated upon enzyme activation [16, 17]. The identified compounds were characterized by moderate potency; however, some of them exhibited up to a tenfold selectivity for IGF1R versus InsR. Based on these results, we concluded that the binding site mentioned above has relevance for designing selective inhibitors of IGF1R. In order to design a IGF1R inhibitor screening set, we created the pharmacophore model (Fig.1) of interaction between the compound series mentioned above and the allosteric site using the available X-ray structure of the IGF1R kinase domain (PDB code 3LWO). The model included a H-donor, a H-acceptor, an aromatic/ pseudo-aromatic ring, as well as any group distanced from the main molecular cluster.

Hydrogen bonding with the carboxyl group of Val1063 is one of the key determinants of binding at the allosteric site. The candidate binder molecule must contain a fragment identifiable as a strong hydrogen bond donor. Ambiguities in the definitions of such donors in various commercially available chemistry search programs led us to establish internal definition criteria for it.

All chemical compounds containing strong hydrogen bond donors were selected from the available compound database of approximately 1,900,000 entries (www.enamine.net) for further filtering. Those included all aliphatic amines, including tertiary amines (which are capable of becoming hydrogen bond donors upon protonation), as well as all other compounds with nonamide and non-sulfonamide NH groups (which were selected using the following SMART string: ([#1][#7;H1] ([!$([#6,#16;X3,X4]=[O])])[!$([#6,#16;X3,X4]=[O])])). All compounds lacking an aromatic ring or an H-acceptor were subsequently removed from the selection. The resulting reduced database (approximately 400,000 compounds) has been further filtered to comply with the created pharmacophore model. All degrees of freedom were allowed for the rotatable bonds, and the additional “forbidden volume” rule was imposed on the protein atoms. Upon processing of the starting database according to the rules described above, 42,031 compounds strictly corresponding to the model criteria were identified. This final filtered set was subjected to a molecular docking study.

Docking was done with the flexible ligand and fixed receptor model, using a systematic docking algorithm (SDOCK+), which demonstrates sufficient ability to reproduce ligand conformations with a minimal rootmean- square deviation (RMSD) with regard to the crystallographic data [18]. The maximum number of computational steps was set at 300, and the 20 best complexes (based on internal QXP scoring functions) were retained for analysis. The binding site model was formed based on the available X-ray data for the complex (3LWO). Amino acid residues with at least some atoms within a 1.0-nm radius around the initial inhibitor were taken into account when designing the binding-site model.

**Fig. 2:**
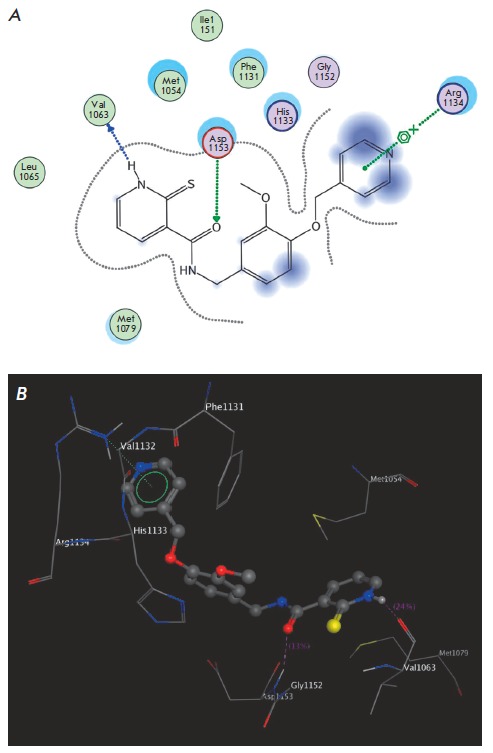
Key interactions of the T2 compound with the IGFR1 binding site model (**A **— two-dimensional diagram showing the key interactions; **B **— ligand-target complex obtained by docking)

Post-docking processing and analysis of the results were performed according to the general logic of the pharmacophore model, which incorporates the key determinants of ligand-site binding strength. The following main geometrical filters were used: hydrogen bonding with Val1063, stacking with Met1054 and Met1079, as well as the secondary filters – electrostatic inter actions with Lys1033 and/or formation of hydrogen bonds with Asp1153 and/or Glu1050, Arg1134 (PDB code 3LW0). The main filters, as well as one or several secondary ones, were always used for selecting compounds. Visual inspection of the automated filtering output was conducted to ensure overall correspondence of the filtering rules to the model. The resulting 1,746 compounds designated as the T(target)-type selection set were submitted to high throughput screening as described below. The interaction between the 1,2-dihydropyridine- 2-thione derivative (compound T2) and the allosteric binding site model is illustrated in Fig. 2 as an example. The compound meets all the basic requirements, and three additional interactions, namely, hydrogen bonding/stacking with Arg1134, His1133, and Asp1154, are possible as well. We consider these factors to be sufficient for inhibitory activity according to the mechanism postulated above.

**Virtual screening – Ligand-based selection**

**Fig. 3:**
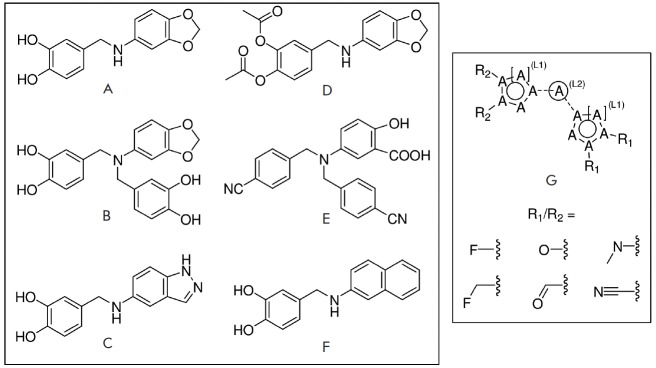
Exemplary IGF1R inhibitors used as a basis of the L-type selection (A–F) and the corresponding Markush formula (G) for analog search

Another productive approach for discovering IGF1R inhibitors was the “ligand-based,” rather than the “target-based,” one; it relied on the published data on IGF1R inhibitors discovered by Levitzky’s group [19]. Since these compounds have been reported to be ATP-noncompetitive and some of them have exhibited substantial selectivity for IGF1R versus InsR kinase, we considered them an attractive starting point for exploring Enamine’s collection in the search for structurally distant novel analogs. A SAR analysis of these active compounds, some of which are shown in Fig. 3, allowed us to identify a number of potentially preferable structural features. In particular, the actives contained 2- or 3-substituted benzene rings linked by saturated NH-CH_2_ or CH_2_-N-CH_2_ linkers; further elongation of the linker by an additional atom decreased potency. At least one group with one hydrogen bond acceptor atom (N or O) must be located at the *para*and/ or *meta*-positions of the linked benzene rings to ensure activity. It was evident that average potency declined for the series: catechols > salicylic acid derivatives > benzodioxols. We hypothesized that the acceptor atoms directly linked to the rings at the *para*- and/or* meta*-positions were the most efficient pharmacophore groups that could be freely rotated to effectively bind to the target site. Additional hydrogen acceptor atoms seemed to provide higher potencies, and a similar level of IGF1R potency was achieved with the H-bond acceptor located either in the condensed aromatic rings or in aliphatic substituents (see Fig. 3A,B,C). Acylation of *para*-/*meta*-hydroxy groups (see Fig. 3A,D) did not significantly change the activity but seemed to have increased selectivity against the insulin kinase receptor and SRC kinase. Fully substituted benzodioxol compounds without hydrogen donors were also active.

These observations were combined in the Markush formula (Fig. 3G). The proposed structure contained at least two 5- or 6- atom aromatic systems, with at least one R-group from the ones listed below at positions 3 and/or 4 of the aromatic system. The R-groups present in the inhibitors described in the literature (i.e. *O*- and *N*-containing substituents), as well as fluoro- and α-fluoroalkyl, were selected as potential H-acceptors. The R-groups were allowed to incorporate the rings. One- to threeatom linkers formed by any nonring bonds (single, double, triple or aromatic) were used to link the aromatic rings. All the atoms in the rings and the linker were set to “any element except hydrogen” type during the database search. A search of Enamine’s ~1.9 million compounds collection *in silico *using the Instant JChem software resulted in the identification of 607 final compounds for HTS selected from 1,327 Markush-compliant compounds after application of the set of medicinal chemistry filters discussed above [14] and setting cutoffs for logP and logS to < 5. This screening selection was designated as the L(ligand)-type compound set.

**High throughput screening and doseresponse measurements**

Screening of the T-type (1647 compounds) and L-type (607 compounds) sets, which were selected as described above, was performed using the commercially available biochemical IGF1R ADP-Glo kinase assay system (Promega Corp.). This assay utilizes a recombinant intracellular kinase domain fragment of IGF1R and is based on quantitation of adenosine diphosphate (ADP), a universal product of any kinase reaction, via enzymatic conversion of ADP to ATP, followed by detection of the luciferase-based luminescent signal [20]. Prior to performing HTS, the assay was validated for enzyme inhibition using the known IGF1R inhibitors — diarylurea derivative PQ401 [21] and tyrphostin AG538 [22], as well as the pan-kinase inhibitor staurosporine. The dose-response curves for all the reference inhibitors were in agreement with the data in the literature. In addition, performance of the HTS assay was tested for day-to-day and plate-to-plate reliability and reproducibility according to the standard HTS guidelines outlined in the Experimental section.

**Fig. 4:**
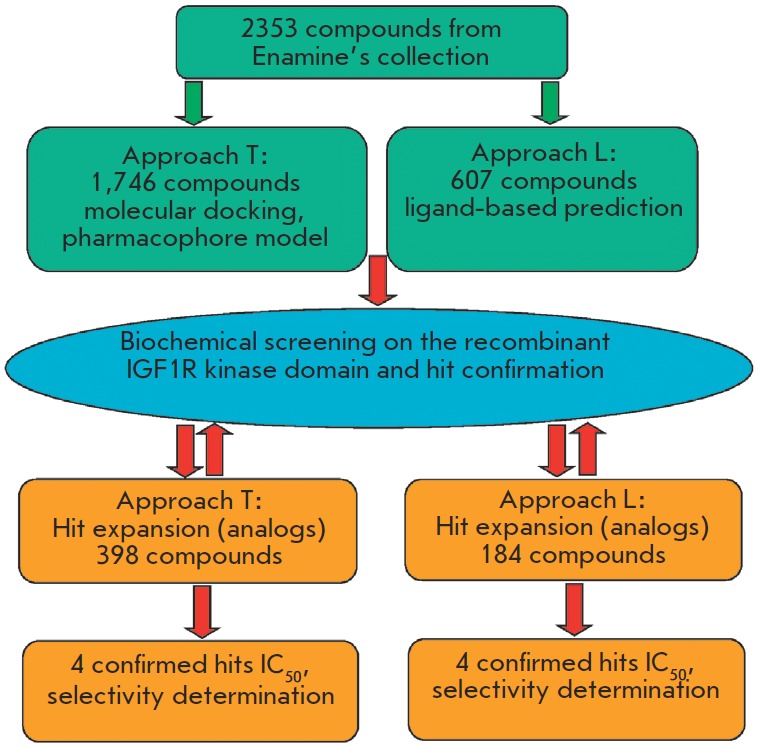
Flowchart of the high-throughput screening procedure

All the compounds exhibiting statistically significant inhibitory activity in the primary high-throughput screening runs (“screening hits”) were re-tested separately at least once. Structural analogs of the confirmed hits were identified in Enamine’s compound repository by chemoinformatics searches, and the resulting sets were additionally screened in the same assay (“hit expansion” screening) as shown in Fig. 4. Hit expansion was done by selecting all the nearest structural relatives of the actives from the collection, whereby all constituent groups in the molecules were subjected to structural variability where possible (sub-structure search). For the T-type compounds, conservation of the key pharmacophores depicted in Fig. 1 was imposed as an additional condition.

Screening of the T-type compound sets in the IGF1R ADP-Glo assay resulted in the identification of 3 confirmed active hits; the fourth hit (L4) was identified after the “hit expansion” screening. Screening of L-type compound sets led to the identification of 3 confirmed hits — L1, L2, and L3; the fourth hit (L4) was also identified after the “hit expansion” screening. These inhibitors were selected from all detected primary screening hits for additional experimental characterization. The selection was based on their estimated potency, reproducibility of the inhibition, and attractive chemical features. In particular, chemical tractability of the inhibitors is facilitated by their structural novelty, potential for synthetic improvements, as well as the absence of undesirable functionalities that might hinder further development of the compound.

**Selectivity of the inhibitors**

Selectivity for the IGF1R of eight hits identified as the result of the HTS campaign and follow-up hit expansion screenings were tested against the insulin receptor (InsR) kinase, as well as the tyrosine kinases Met, Syk, and BTK. While InsR is the closest evolutionary relative of IGF1R, the other 3 tyrosine kinases are more distantly related receptor-type (Met) or cytoplasmic (Syk, BTK) tyrosine kinases. When comparing IGF1R and InsR, the IC_50_ values were measured for the inhibitors (Table 1), while the inhibition of the remaining kinases was evaluated at a single concentration point. All the kinase assays were run under experimental conditions similar to those of the IGF1R assay, with regard to the kinetic parameters of kinase reactions, and were read using the same commercial ADP detection system ADP-Glo (Promega Corporation) in order to ensure maximum uniformity for comparing the inhibition degrees. The small kinase panel used in this study cannot provide a comprehensive profile of the kinase selectivity of the tested inhibitors; however, it offers a general indication of the selectivity for the IGF1R target within the most closely evolutionarily related tyrosine kinase subfamily of over 500 human protein kinases. The data (Table 1) indicates limited selectivity of the compounds between IGF1R and InsR kinases, with some of the compounds being essentially nonselective (L1, L3, L4), while the others reproducibly exhibited 1.5-4-fold selectivity for IGF1R versus InsR (L2, T2, T4). Interestingly, compounds T1 and T3 exhibited a substantially stronger (5-10-fold) inhibition of InsR versus IGF1R in our experiments. This selectivity was similar or higher than that of almost all the known small molecule inhibitors of IGF1R demonstrated in a valid biochemical assay [23], apparently reflecting a very high degree of similarity between the receptors at their catalytic sites and in their close vicinity. The single concentration data on the inhibition of the more distantly related tyrosine kinases Met, BTK, and Syk (Table 2) suggests no inhibition (compounds L1, T3) or weak inhibition for some of these kinases, with estimated IC_50_ values in excess of 50 μM.

**Table 1:** Inhibition of IGF1R and InsR (IC^50^) by L- and Tseries hit compounds.

Compound	Structure	IC^50^, μM	
		IGF1R	InsR
L1	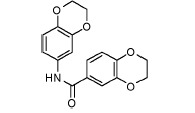	18	22
L2	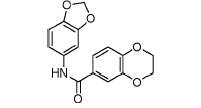	25	100
L3	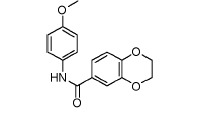	26	29
L4	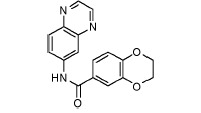	25	30
T1	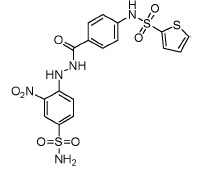	~100	20
T2	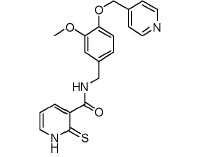	18	30
T3	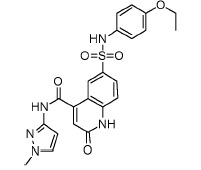	~100	10
T4	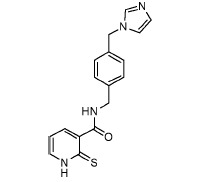	7	10

**Table 2:** Inhibition of Met, Syk and BTK kinases by L- and T-series hit compounds.

Compound*	Activity Met, %	±SD	Activity Syk, %	±SD	Activity Btk, %	±SD
L1	102	3	109	16	98	10
L2	63	2	48	5	106	6
L3	77	4	88	4	106	8
L4	60	2	53	9	113	11
T1	78	2	84	10	92	11
T2	62	4	72	13	83	8
T3	98	3	115	12	81	8
T4	75	3	115	19	82	5

*Concentration of compounds is 40 μM.

**Mechanistic kinetic studies**

**Fig. 5:**
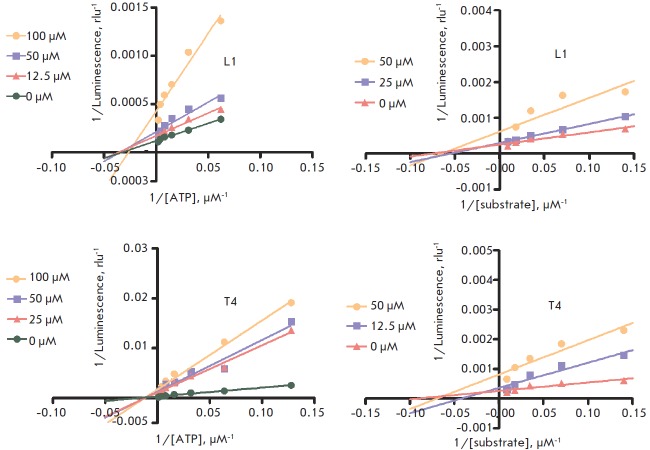
Lineweaver-Burk plots for compounds L1 and T4

Two arbitrarily selected compounds representing both series (L1 and T4) were used in the experiments of competitive inhibition kinetics with IGF1R kinase to investigate the inhibition mechanism (Fig. 5). The Lineweaver- Burk plot analysis indicates that both compounds exhibit non-competitive inhibition with regard to both ATP and the substrate. This experimental conclusion is consistent with the rationales used for the virtual selection of compounds for HTS and suggests an allosteric inhibition mode of IGF1R kinase by both the L- and T-type compounds. The binding site for T-type compounds is likely to align with the allosteric site defined for the prototypic indolealkylamines that were used to establish our pharmacophore model [15] and is spatially separated from the enzyme catalytic site. In the case of L-type compounds, localization of their putative binding site(s) on the kinase domain fragment is unclear. Some prototypic compounds used for our modeling have also been reported to be noncompetitive with ATP [19]; however, no substrate competition or extensive molecular modeling data are available. Due to the generally higher variability of the kinase domain regions distant from the conserved active sites, the allosteric mode of binding has more potential for fine-tuning the selectivity profiles of the inhibitors during their synthetic optimization stage.

Therefore, the set of IGF1R inhibitors described above meets the conventional requirements imposed on high-throughput screening hits: reproducibility and dose-dependence of the pharmacological response, acceptable potency of the molecular target inhibition (IC_50_=10–25 μM) and selectivity versus related targets, absence of structural elements undesirable from a medicinal chemical perspective, novelty of the compounds, and availability of synthetic routes for their modification. In addition, the compounds exhibit allosteric inhibitor properties, which was one of the objectives of the project. In conclusion, the preliminary characterization of the two inhibitor series identified in the course of the screening campaign suggests that these compounds can serve as attractive starting points for a medicinal chemistry optimization towards novel, small molecule therapeutics targeting IGF1R.

## Abbreviations

IGF1R: insulin-like growth factor type 1 receptor
InsR: insulin receptor
RTK: receptor tyrosine kinase
TKI: tyrosine kinase inhibitor
HTS: high-throughput screening
ATP: adenosine triphosphate
ADP: adenosine diphosphate
